# A Pilot Trial Using Telemedicine in Radiation Oncology: The Future of Health Care Is Virtual

**DOI:** 10.1089/tmr.2021.0007

**Published:** 2021-06-14

**Authors:** Ryan C. Miller, Brittany A. Simone, Joseph F. Lombardo, James Taylor, Kamila Nowak-Choi, Kevin Ko, Linda Ferguson, Ann Donnelly, Ayesha S. Ali, Wenyin Shi, Adam P. Dicker, Nicole L. Simone

**Affiliations:** ^1^ Department of Radiation Oncology, Sidney Kimmel Cancer Center at Thomas Jefferson University, Philadelphia, Pennsylvania, USA.; ^2^ Department of Radiation Oncology, Upstate Cancer Center, SUNY Upstate Medical University, Syracuse, New York, USA.

**Keywords:** telemedicine, radiation oncology, e-health, patient satisfaction, provider satisfaction

## Abstract

**Background:** Social determinants of health directly affect cancer survival. Driven by advances in technology and recent demands due to COVID-19, telemedicine has the ability to improve patient access to care, lower health care costs, and increase workflow efficiency. The role of telemedicine in radiation oncology is not established.

**Materials and Methods:** We conducted an IRB-approved pilot trial using a telehealth platform for the first post-radiation visit in patients with any cancer diagnosis. The primary endpoint was feasibility of using telehealth defined by completion of five telehealth visits per month in a single department. Secondary endpoints included the ability to assess patients appropriately, patient and physician satisfaction. Physicians were surveyed again during the pandemic to determine whether viewpoints changed.

**Results:** Between May 27, 2016 and August 1, 2018, 37 patients were enrolled in the Telehealth in Post-operative Radiation Therapy (TelePORT) trial, with 24 evaluable patients who completed their scheduled telehealth visit. On average, 1.4 patients were accrued per month. All patients were satisfied with their care, had enough time with their physician and 85.7% believed the telehealth communication was excellent. All physicians were able to accurately assess the patient's symptoms via telehealth, whereas 82.3% felt they could accurately assess treatment-related toxicity. Physicians assessing skin toxicity from breast radiation were those who did not feel they were able to assess toxicity.

**Discussion and Conclusions:** Both health care providers and patients have identified telemedicine as a suitable platform for radiation oncology visits. Although there are limitations, telemedicine has significant potential for increasing access of cancer care delivery in radiation oncology.

## Introduction

Creative solutions to increase patients' access to health care have begun to emerge. Telemedicine, which refers to the use of communications technology to deliver health care services to patients who may be limited in their ability to have office-based visits, is a potential solution.^1^ Implementing technology platforms may eliminate the need for some patients to be physically present in clinic. Interest in the use of telemedicine has steadily grown due to the potential to lower health care costs, improve patient access to care, and increase clinic efficiency. However, the role of telemedicine in radiation oncology has not been established.^1,2^

A significant number of cancer patients will undergo radiation therapy as part of their care plan. Radiation can be physically demanding since patients generally undergo several weeks of daily treatment. Patients routinely have post-treatment follow-up 4–8 weeks after radiation to ensure they are treated for potential radiation toxicity, to provide psychological support and information about next steps in their care.^3,4^ In-person post-treatment visits have come under scrutiny due to limited resources and therefore, this may lend itself as an ideal place for telemedicine.^5–7^

Telemedicine eligible patients may not require in-depth physical exams for each visit. For cancers including breast, colorectal, and testicular, physical examination has not been shown to significantly contribute to the detection of recurrence.^8–10^ A number of studies have shown that telephone follow-up can be as effective as face-to-face visits in addressing psychosocial effects of disease and treatment.^11,12^ Nurses who conducted telephone follow-up for early stage breast cancer patients expressed confidence in their ability to deliver care through telemedicine platforms.^12^

The feasibility of remote follow-up after treatment has been shown in high-grade glioma, breast, prostate, bladder, endometrial, and colorectal cancers.^13–18^ Patients reported higher levels of satisfaction with telephone follow-up compared with traditional outpatient services with no impairment in recurrence detection. Patients prefer remote follow-ups due to convenience, reduced travel and wait times, and lower cost.^2,12^ In another study, Shaida et al. examined patient satisfaction with nurse-led telephone follow-up for patients with prostate cancer.^15^ They found no significant differences in general satisfaction or perception of professional care between telephone and outpatient consultations. However, patients who received telephone consultations were less satisfied regarding the depth of relationship with their provider and the perceived time of visit, thus suggesting some limitations with the use of technology.

Although evidence is promising, the majority of studies examining telemedicine are not randomized, have small patient numbers, and do not use validated patient satisfaction metrics. Our study aims at examining both patient and physician satisfaction with follow-up care using validated tools. We look at the implementation of telemedicine follow-up in patients who have undergone radiotherapy, focusing on the first post-treatment visit. Given more widespread adoption for new patient and follow-up visits with changes in Medicare guidelines, we sought to determine whether health care providers were satisfied with the telemedicine platform during the COVID-19 pandemic to provide a more robust data set. It is important to understand satisfaction with these visits given how vital telemedicine has become for current practice.

## Materials and Methods

We conducted a single-institution feasibility study using telehealth for patients who had received radiation therapy for any cancer diagnosis. The protocol was approved by the IRB. All patients provided informed consent.

### Patients

All patients treated in the Radiation Oncology Department at the Sidney Kimmel Cancer Center at Thomas Jefferson University were screened at their last on-treatment visit for eligibility for the trial. Eligible patients were older than 18 years of age, had a Karnofsky Performance Score of greater than or equal to 60, and had completed a course of radiation therapy. Patients had to be able to complete questionnaires in English and were required to have access to a device, such as a computer, smartphone, or tablet, and a convenient Internet connection for their telemedicine visit. Patients had to complete their telehealth visit from a location where their provider was licensed to practice medicine.

### Telemedicine visit

Patients were required to complete their single telemedicine visit within 8 weeks of completing radiation. Initially, an in-house software termed JeffConnect (developed at Thomas Jefferson University in conjunction with Teladoc Health, Inc., Purchase, NY) was used to complete telemedicine visits and after November 26, 2016 the Epic telecommunication software available through the MyChart feature of Epic (Epic Systems Corporation, Verona, WI) was used.

### Questionnaires

Patients were asked to respond to three validated questionnaires within 4 weeks of completing the telemedicine visit: the 18-item Patient Satisfaction with Cancer Care (PSCC) questionnaire, the 14-item Communication Assessment Tool (CAT), and the 10-item Health Care System Distrust Scale (HSDS). The PSCC addresses multiple domains of care, including access/logistics, interpersonal skills of the provider, information gathering and reporting, and coordination of care. Each question is answered on a Likert scale (1–5), where 1 = “strongly agree,” and 5 = “strongly disagree.” Lower scores indicate a higher satisfaction with cancer care. The CAT addresses patient satisfaction with key aspects of provider communication and interpersonal skills, and items are scored on a Likert scale (1–5) where 1 = “poor” and 5 = “excellent.” A higher score indicates better communication skills. The HSDS is designed to assess the belief that providers would act in a patient's best interest to prevent a potentially negative outcome. Items are scored on a Likert scale (1–5), where 1 = “strongly disagree” and 5 = “strongly agree.”

Radiation oncology providers completed a 19-item survey, designed within the institution, regarding the usability and efficacy of the telemedicine platform. The questionnaire determined whether the provider could evaluate the patients' radiation toxicity, adequately review patient results, give appropriate future instructions and if they felt the patient needed further evaluation in person. The encounter duration was also noted.

### COVID-19 pandemic provider reassessment

Due to increased utilization of telemedicine brought on by the COVID-19 pandemic, providers were asked to complete the same 19-item response form discussed earlier for new patient, follow-up, and first post-radiation encounters. These surveys were distributed from April 1st, 2020 to April 30th, 2020.

### Statistical design

The primary endpoint of this study was the number of patients enrolled per month to determine the feasibility of conducting a future non-inferiority randomized clinical trial comparing telemedicine and in-person visits. For that non-inferiority trial, we projected that we would need to enroll a total of ∼180 patients in 3 years (i.e., a minimum accrual rate of 5 patients per month). If the true accrual rate is 6 patients per month (i.e., 72 patients per year), then the study has 83% power to establish that the accrual rate exceeds 5 patients per month (using a 1-sided test for Poisson rate, with alpha 0.05).

Secondary endpoints included patient satisfaction as measured by the PSCC, assessment of communication as measured by the CAT, patient distrust in the health care system as measured by the HSDS, physician satisfaction as measured by the modified physician satisfaction scale, and ability to assess patients appropriately as measured by the physician questionnaire. With an expected accrual of 60 patients, this study had 90% power to establish that patient satisfaction will exceed 80%, assuming a true PSCC score of about 90% and a standard deviation of about 5% (using a one-sample *t*-test with alpha 0.05).

## Results

### Patient accrual and visit completion

**FIG. 1.**
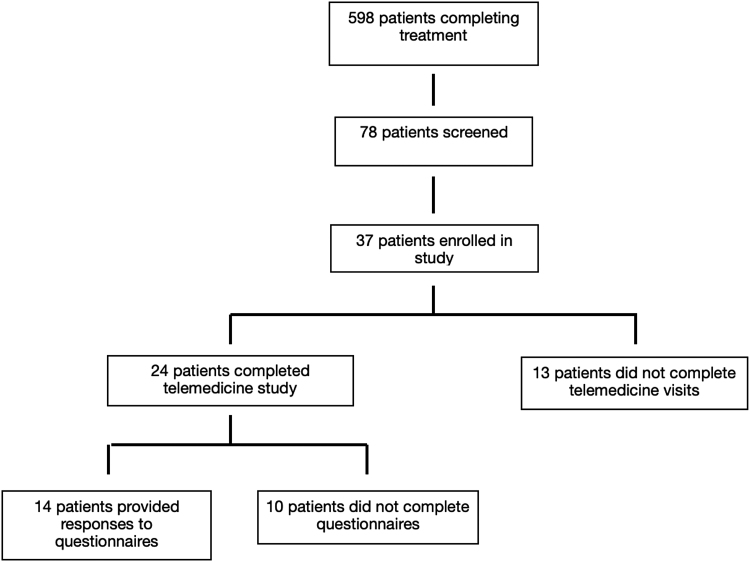
Patient accrual and visit completion. During the time that the study was open, a total of 598 patients completed radiation, 78 patients screened, and 37 patients were enrolled in the trial based on eligibility criteria. Of the 37 enrolled patients, 24 completed a telemedicine visit and 14 completed the questionnaire. The average number of patients accrued per month over the time period that the study was open for accrual was 1.4 patients.

Between May 27, 2016 and August 1, 2018, 37 patients were enrolled in the trial based on eligibility criteria (Fig. 1). A total of 15 men and 22 women were enrolled (Table 1). Of those enrolled, there were: 1 Asian (2.7%), 11 Black non-Hispanic (29.7%), 23 White non-Hispanic (62.1%), and 2 Hispanic patients enrolled (5.4%). A total of 23 patients (62%) were younger than age 65. Patients with many cancer subtypes were enrolled, including 27% with breast, 19% with prostate, and 19% with CNS cancer. Most patients (51%) were college graduates. Of the 37 enrolled patients, 24 completed a telemedicine visit, and of this cohort, 14 completed the questionnaire. The average number of patients accrued per month over the accrual period was 1.4 patients, which did not meet the previously defined enrollment of 5 patients per month.

**Table 1.** Patient Demographics

Characteristic	% of patients (*n*)
Age	
<65 years	62 (23)
>65 years	38 (14)
Sex	
Male	41 (15)
Female	59 (22)
Type of malignancy	
Breast	27 (10)
Prostate	19 (7)
CNS	19 (7)
Skin	11 (4)
Lung	8 (3)
Hepatobiliary	8 (3)
Gynecologic	8 (3)
Highest level of education	
Less than high school	11 (4)
High school or GED	27 (10)
College	51 (19)
Master's or doctorate	11 (4)
Ethnicity	
Asian	2.7 (1)
Black non-Hispanic	29.7 (11)
White non-Hispanic	62.1 (23)
Hispanic	5.4 (2)

A total of 37 patients were enrolled on the trial, including 15 men (41%) and 22 women (59%). In regards to age, 23 patients were younger than age 65 (62%). Patients with various cancer subtypes were included, with breast (27%), prostate (19%), and CNS (19%) making up the highest proportions. Most patients were college graduates (51%). White non-Hispanic patients (62.1%) and Black non-Hispanic patients (29.7%) were the most represented ethnicities on the trial.

CNS, central nervous system; GED, general educational development test.

### Patient satisfaction

**FIG. 2.**
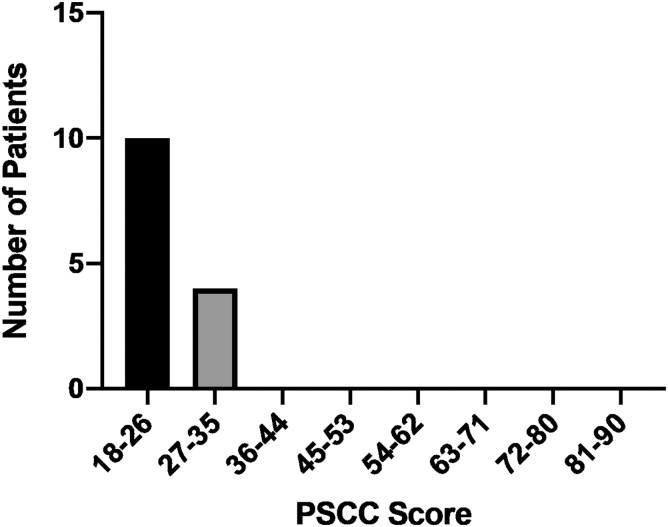
PSCC questionnaire score. The PSCC is an 18-item questionnaire that addresses multiple domains, including: access/logistics, interpersonal skills of the provider, information gathering and reporting, and coordination of care on a Likert scale. The PSCC questionnaire has a score range between a minimum of 18 (best score) and a maximum of 90 (worst score). In this cohort, the scores ranged from 18 to 32 with a mean of 22.6 and a median of 21. PSCC, Patient Satisfaction with Cancer Care.

The PSCC scale was completed by 14 patients. The PSCC questionnaire has a score range between a minimum of 18 (best score) and a maximum of 90 (worst score), with this cohort having scores ranging from 18 to 32 with a mean of 22.6 and a median of 21 (Fig. 2), which indicates that these patients are highly satisfied with their cancer care via telemedicine. All patients either “strongly agree” or “agree” that their questions were answered, scheduling appointments was easy, and they had enough time with their doctor.

### Patient assessment of communication

**FIG. 3.**
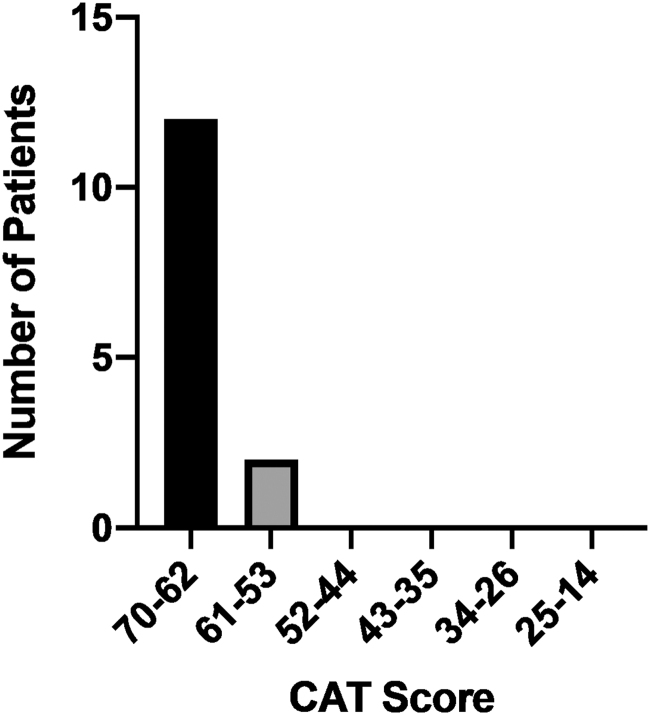
CAT questionnaire score. The CAT is a 14-item questionnaire that addresses patient satisfaction with key aspects of provider communication and interpersonal skills, and items are scored on a Likert scale. The CAT questionnaire has a score range between a minimum of 14 (worst score) and a maximum of 70 (best score). In this cohort, the scores ranged from 55 to 70 with a mean of 67.5 and a median of 70. CAT, Communication Assessment Tool.

The CAT indicated that all 14 patients were satisfied with the communication they had with their provider via telehealth. The score ranges from a minimum of 14 (worst score) to a maximum of 70 (best score). In this cohort, the scores ranged from 55 to 70 with a mean of 67.5 and a median of 70 (Fig. 3), indicating satisfaction. All patients (100%) responded “excellent” or “very good” when answering all of the following statements: “Greeted me in a way that made me feel comfortable, treated me with respect, paid attention to me, let me talk without interruptions, checked to be sure I understood everything, involved me in decisions as much as I wanted, showed care and concern and spent the right amount of time with me.” No patients (0%) responded “poor” or “fair” to any of the 14 items.

### Health care system distrust

To assess a separate outcome from satisfaction, health care system trust was evaluated by patients completing their telehealth encounter. The HSDS was completed by 14 patients and demonstrated approximately one-quarter of the patients to have a fair bit of distrust in medical care noted by consistent responses throughout the questions. For example, 4 patients (28.5%) thought that “when they take my blood, they do tests they don't tell me about,” 3 patients (21.4%) were not sure whether “medical experiments could be performed on them without their knowledge,” 7 patients (50%) were either not sure or agreed that “the health care system cares more about holding costs down than it does about doing what is needed for my health,” and 2 patients (14.2%) were unsure or believed that their medical records were not kept private. However, in response to the statement, “I receive high-quality medical care from the health care system,” 13 patients (92.9%) “strongly agree” or “agree” and 1 patient (7.1%) was “not sure,” suggesting that the relationship that patients build with their direct provider is the most important indicator of satisfaction with the health care system even when adapting to telehealth.

### Health care provider satisfaction with telemedicine during the first post-treatment visit

**FIG. 4.**
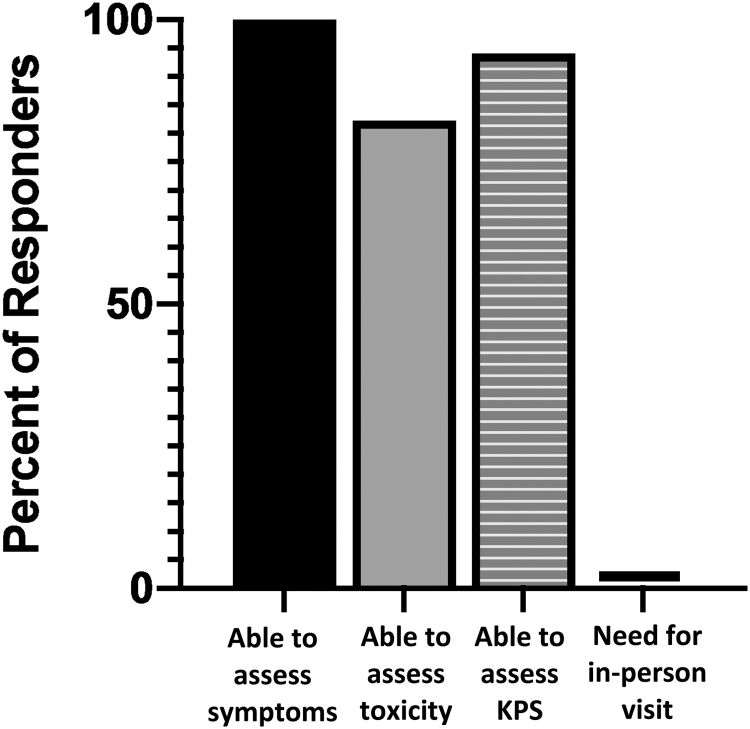
Provider assessment of telemedicine platform. A total of 11 providers participated during the initial enrollment period, and a total of 17 responses were recorded (some providers submitted multiple entries). During the initial enrollment when only first post-treatment visits were being conducted via telemedicine, all providers (100%) felt that they were able to accurately assess the patient's symptoms via the platform, whereas 14 (82.3%) of the respondents felt that they could accurately assess treatment-related toxicity. Sixteen (94.1%) of the respondents were able to document the patient's KPS, and no provider (0%) required their patient to come in for a subsequent in-person encounter. KPS, Karnofsky Performance Status.

Nine physicians and 2 nurse practitioners participated during the initial enrollment period, and completed 17 responses for the visits they performed (some providers submitted multiple entries). During the initial enrollment when only first post-treatment visits were being conducted via telemedicine, all providers (100%) felt that they were able to elicit adequate history from the patient, accurately assess the patient's symptoms via the platform, and document an accurate Karnofsky Performance Status, whereas 82.3% of the respondents felt that they could accurately assess treatment-related toxicity (Fig. 4). Providers who were assessing patients with resolving acute toxicity from breast radiation were the two who did not believe they could accurately assess toxicity. No provider (0%) felt the need to bring their patient in for an additional face-to-face visit after the telemedicine visit, and 100% were satisfied that they were able to communicate facts adequately to the patient and give the patient further instructions. Encounters ranged from 10 to 30 min, with 10 min cited as the most frequent duration (95% CI, 14.5 ± 2.7 min). Only one respondent (5.8%) felt that they would not use the telemedicine system again. Fourteen respondents (82.3%) “strongly disagree” or “disagree” that the telemedicine system was frustrating to use, whereas 2 (11.8%) were “not sure” and 1 (5.8%) “agreed.”

### Health care provider satisfaction with telemedicine during the COVID-19 pandemic

During March 2020, when COVID-19 was declared a pandemic by the World Health Organization (WHO), a total of 91 telemedicine encounters were conducted in the department, including new patients and follow-ups. Providers were again asked to evaluate their satisfaction with the telehealth visit, and 23 provider responses were recorded: 12 responses recorded satisfaction with new patient visits, 10 for follow-ups, and 1 for a first post-treatment visit. All providers (100%) felt that they were able to accurately assess patients' symptoms via the telemedicine platform, and 19 respondents (82.6%) did not feel the need to bring in the patient for further evaluation. All providers (100%) either “strongly agreed” or “agreed” that they felt patients were satisfied with the telemedicine encounter. Compared with an in-person encounter, 18 respondents (78.2%) “strongly disagreed” or “disagreed” that they felt they were not getting all the information they needed. All providers (100%) “strongly agreed” or “agreed” that they would use the telemedicine platform again.

## Discussion

The COVID-19 pandemic has highlighted the need for optimizing cancer care delivery by making it accessible to people encountering different circumstances.^19^ To our knowledge, this is the first study to examine the role of telemedicine as a platform for post-treatment follow-up visits in radiation oncology using validated tools, as well as the first study to assess satisfaction with the platform in conducting radiation oncology encounters during the COVID-19 pandemic. All patients enrolled on this study either “strongly agreed” or “agreed” with regards to being satisfied with their telemedicine visit. Although all physicians determined that they were able to adequately assess the patients' symptoms, 82.3% believed that they were able to assess toxicity during the first post-treatment visit.

A radiation oncology patient's desire to participate in a telemedicine visit may be enticing for some but may pose challenges for others. In regards to study limitations, our study enrolled a total of 37 patients, with only 24 completing the visit. Unfortunately, our study did not meet the accrual goal during the enrollment period. We found that radiation patients were initially reluctant to the idea of a telemedicine encounter. Since these patients are seen by several members of the radiation oncology team on a daily basis for several weeks, the bond they have formed during that time creates a desire for an in-person visit. It is important to note that despite the reluctance or significant number of patients noted to have distrust of the health care system on this study, they still seemed to indicate their global satisfaction with the telehealth platform. This may suggest that the bond and trust a patient and their provider build together may allow for adaptation in this space. During this trial, poor accrual can be attributed, in part, to our institution's transition of the electronic medical record, which placed the study on hold and therefore prevented the study from meeting the expected number of patients enrolled per month.

Digital health literacy is an important component in expanding the world of telemedicine care delivery. We found that the majority of patients who enrolled in our study were younger than 65 and had a college degree or higher. Within the United States, digital barriers often affect those who are older, from rural communities, come from racial/ethnic minority populations, have limited English proficiency, or have low socioeconomic status.^20–22^ For example, low-income individuals in the United States have ∼71% smartphone ownership, 82% use Internet, 53% have basic digital health literacy, and 59% have home broadband access.^20,23,24^ One in four Americans may not have the digital literacy or access to a device to have a telemedicine visit.^23,24^ An easy-to-use telemedicine platform may be crucial to its success. Once our study transitioned to using the MyChart system in Epic, completion of telemedicine visits became simplified and much more attractive to patients. In the year after the transition to the Epic system, a total of 111 telemedicine visits were scheduled. An additional limitation of the study that affected enrollment was including only those patients completing their first post-treatment follow-up visit. This time point was chosen, because there is a positive financial implication in terms of resource utilization and improved clinic workflow if these visits are not performed in the clinic. In addition, the initial post-treatment visit is bundled by insurance companies to be part of the radiation treatment fee and therefore does not carry a charge to the patient. Additional obstacles to accrual included mixed support during the enrollment period from faculty, as there was no formalized training on how to use the platform, scheduling conflicts, and inability for some providers to conduct the full physical examination necessary to guide management.

It is important to note that during the COVID-19 pandemic, the number of encounters increased significantly to 91 during the month of March 2020 (new patient, follow-ups, and first post-treatment visits). This is in line with other institutions, including a health system in New York that reported that within 2 weeks of requiring the majority of attending physicians to work remotely, more than 90% of weekly on-treatment visits were being performed via a telemedicine platform.^25^ Our study demonstrated that providers found the telemedicine platform as a suitable alternative to in-person visits, both for post-treatment visits during the initial enrollment and for new patient encounters during the COVID-19 pandemic. Future studies should aim at exploring whether patients, too, are satisfied with telemedicine encounters for their initial consultation visits, as this study only assessed their attitudes with the first post-treatment encounter during the enrollment period.

The use of a telemedicine platform for decongestion of the clinic has positive implications in regards to patient satisfaction, health care spending, and patient safety. It has been implemented in both primary care and specialty settings, with promising results.^1,2,26^ Patients save both time and money by utilizing this resource. Moreover, the possible implementation of telemedicine in clinical trials could improve both patient access to trials and clinical trial data integrity.^27^ The use of a telemedicine platform can significantly improve access to care for patients who reside in a rural setting and who do not have direct access to a tertiary care center.^28^ Our institution covers a wide patient catchment area, and patients may drive several hours to be present in clinic. This is associated with significant cost for travel, as well as time taken out of work. Telemedicine offers an excellent method for reducing the costs associated with oncologic care and should be examined as an alternative to traditional face-to-face clinic visits in expanded settings. Lastly, telemedicine can minimize exposure risk for patients with cancer, who may be immunocompromised secondary to their disease process or oncologic treatments and are at greater risk for severe COVID-19 infection.^29^

## Conclusions

Although some patients strongly prefer to see their radiation oncologist in person, and although there are certain limitations in regards to conducting an adequate physical exam to gauge treatment-related toxicity, our study has demonstrated that both providers and patients feel that it is an adequate modality in a multitude of settings, including new, follow-up, and first post-radiation treatment. From our results, patients were satisfied with the telemedicine encounter, providers were able to evaluate patient symptoms adequately, and telemedicine did not increase health care distrust. Telemedicine is a platform that has significant potential for the field of radiation oncology and should be strongly considered for patients when appropriate.

## Abbreviations Used

CAT: Communication Assessment Tool
CNS: Central Nervous System
GED: General Educational Development Test
HSDS: Health Care System Distrust Scale
NCI: National Cancer Institute
PSCC: Patient Satisfaction with Cancer Care
WHO: World Health Organization

## References

[1] Currell R, Urquhart C, Wainwright P, et al. Telemedicine versus face to face patient care: effects on professional practice and health care outcomes. Nurs Times 2001;97:35 11957594

[2] Mair F, Whitten P. Systematic review of studies of patient satisfaction with telemedicine. BMJ (Clin Res Ed) 2000;320:1517–1520 10.1136/bmj.320.7248.1517 PMC27397 10834899

[3] Ataman OU, Barrett A, Davidson S, et al. Audit of effectiveness of routine follow-up clinics after radiotherapy for cancer: a report of the react working group of ESTRO. Radiother Oncol 2004;73:237–249 15542172 10.1016/j.radonc.2004.05.001

[4] van Hezewijk M, Ranke GM, van Nes JG, et al. Patients' needs and preferences in routine follow-up for early breast cancer; an evaluation of the changing role of the nurse practitioner. Eur J Surg Oncol 2011;37:765–773 21764242 10.1016/j.ejso.2011.06.007

[5] James ND, Guerrero D, Brada M. Who should follow up cancer patients? Nurse specialist based outpatient care and the introduction of a phone clinic system. Clin Oncology (R Coll Radiol (Great Britain)) 1994;6:283–287 10.1016/s0936-6555(05)80267-5 7826919

[6] Brada M. Is there a need to follow-up cancer patients? Eur J Cancer (Oxford, England: 1990) 1995;31a:655–657 10.1016/0959-8049(95)00079-x 7640033

[7] Teagle A, Gilbert DC. Remote follow-up strategies after cancer treatment: a lot of opportunities. Clin Oncol (R Coll Radiol (Great Britain)) 2014;26:622–624 10.1016/j.clon.2014.05.009 24969682

[8] Jeffery M, Hickey BE, Hider PN, et al. Follow-up strategies for patients treated for non-metastatic colorectal cancer. Cochrane Database Syst Rev 2016;11:Cd002200 27884041 10.1002/14651858.CD002200.pub3 PMC6464536

[9] Montgomery DA, Krupa K, Cooke TG. Follow-up in breast cancer: does routine clinical examination improve outcome? A systematic review of the literature. Br J Cancer 2007;97:1632–1641 18000508 10.1038/sj.bjc.6604065 PMC2360278

[10] Cunniffe NG, Robson J, Mazhar D, et al. Clinical examination does not assist in the detection of systemic relapse of testicular germ cell tumour. Clin Oncol (R Coll Radiol (Great Britain)) 2012;24:39–42 10.1016/j.clon.2011.06.002 21723715

[11] Gotay CC, Bottomley A. Providing psycho-social support by telephone: what is its potential in cancer patients? Eur J Cancer Care 1998;7:225–231 10.1046/j.1365-2354.1998.00110.x 9919109

[12] Beaver K, Williamson S, Chalmers K. Telephone follow-up after treatment for breast cancer: views and experiences of patients and specialist breast care nurses. J Clin Nurs 2010;19:2916–2924 20649914 10.1111/j.1365-2702.2010.03197.x

[13] Sardell S, Sharpe G, Ashley S, et al. Evaluation of a nurse-led telephone clinic in the follow-up of patients with malignant glioma. Clin Oncol (R Coll Radiol (Great Britain)) 2000;12:36–41 10.1053/clon.2000.9108 10749018

[14] Faithfull S, Corner J, Meyer L, et al. Evaluation of nurse-led follow up for patients undergoing pelvic radiotherapy. Br J Cancer 2001;85:1853–1864 11747326 10.1054/bjoc.2001.2173 PMC2364007

[15] Shaida N, Jones C, Ravindranath N, et al. Patient satisfaction with nurse-led telephone consultation for the follow-up of patients with prostate cancer. Prostate Cancer Prostatic Dis 2007;10:369–373 17353916 10.1038/sj.pcan.4500958

[16] Beaver K, Tysver-Robinson D, Campbell M, et al. Comparing hospital and telephone follow-up after treatment for breast cancer: randomised equivalence trial. BMJ (Clin Res Ed) 2009;338:a3147 10.1136/bmj.a3147 PMC2628299 19147478

[17] Kimman ML, Bloebaum MM, Dirksen CD, et al. Patient satisfaction with nurse-led telephone follow-up after curative treatment for breast cancer. BMC Cancer 2010;10:174 20429948 10.1186/1471-2407-10-174 PMC2880988

[18] Smits A, Lopes A, Das N, et al. Nurse-led telephone follow-up: improving options for women with endometrial cancer. Cancer Nurs 2015;38:232–238 25098923 10.1097/NCC.0000000000000177

[19] Wright JH, Caudill R. Remote treatment delivery in response to the COVID-19 pandemic. Psychother Psychosom 2020;89:130–132 32213775 10.1159/000507376 PMC7179517

[20] San Francisco Digital Equity. Digital Equity. San Francisco: San Francisco Mayor's Office of Housing and Community Development, 2018

[21] Tsai HS, Shillair R, Cotten SR. Social support and “playing around”: an examination of how older adults acquire digital literacy with tablet computers. J Appl Gerontol 2017;36:29–55 26491029 10.1177/0733464815609440 PMC5505553

[22] Nouri SS, Avila-Garcia P, Cemballi AG, et al. Assessing mobile phone digital literacy and engagement in user-centered design in a diverse, safety-net population: mixed methods study. JMIR Mhealth Uhealth 2019;7:e14250 31469083 10.2196/14250 PMC6740160

[23] Pew Research Center. Demographics of Mobile Device Ownership and Adoption in the United States, Washington, DC, 2018

[24] Pew Research Center. Demographics of Internet and Home Broadband Usage in the United States, Washington, DC, 2019

[25] Buckstein M, Skubish S, Smith K, et al. Experiencing the surge: report from a large New York radiation oncology department during the COVID-19 Pandemic. Adv Radiat Oncol 2020;5:610–616 32377596 10.1016/j.adro.2020.04.014 PMC7199705

[26] Polinski JM, Barker T, Gagliano N. Patients' satisfaction with and preference for telehealth visits. J Gen Intern Med 2016;31:269–275 26269131 10.1007/s11606-015-3489-x PMC4762824

[27] Galsky MD, Stensland KD, McBride RB. Geographic accessibility to clinical trials for advanced cancer in the United States. JAMA Int Med 2015;175:293–295 10.1001/jamainternmed.2014.6300 25437434

[28] Dickinson R, Hall S, Sinclair JE, et al. Using technology to deliver cancer follow-up: a systematic review. BMC Cancer 2014;14:311 24885758 10.1186/1471-2407-14-311 PMC4101828

[29] Zhang L, Zhu F, Xie L. Clinical characteristics of COVID-19-infected cancer patients: a retrospective case study in three hospitals within Wuhan, China. Ann Oncol 2020;31:894–901 32224151 10.1016/j.annonc.2020.03.296 PMC7270947

